# Effect of Behavioral Change Communication and Livestock Feed Intervention on Dietary Practices in a Kenyan Pastoral Community: A Randomized Controlled Trial

**DOI:** 10.3390/nu17182997

**Published:** 2025-09-19

**Authors:** Nyamai Mutono, Josphat Muema, Zipporah Bukania, Irene Kimani, Erin Boyd, Immaculate Mutua, George Gacharamu, Francis Wambua, Anita Makori, Joseph Njuguna, Christine Jost, Abdal Monium Osman, Darana Souza, Guy H. Palmer, Jonathan Yoder, S. M. Thumbi

**Affiliations:** 1 Paul G. Allen School for Global Health, Washington State University, Pullman, WA 99164, USA; gpalmer@wsu.edu (G.H.P.); thumbi.mwangi@wsu.edu (S.M.T.); 2 Center for Epidemiological Modelling and Analysis, University of Nairobi, Nairobi P.O. Box 19676-00202, Kenya; anitamakori99@gmail.com; 3 Centre for Public Health, Kenya Medical Research Institute, Nairobi P.O. Box 19464-00202, Kenya; zbukania@gmail.com; 4 Food and Agriculture Organization of the United Nations, Nairobi P.O. Box 30470, Kenya; irene.kimani@fao.org (I.K.); joseph.njuguna@fao.org (J.N.); 5 United States Agency for International Development’s Bureau for Humanitarian Assistance (USAID/BHA), Washington, DC 20523, USA; 6 Ministry of Health, Government of Marsabit County, Marsabit 60500, Kenya; immpr14@gmail.com; 7 United Nations Children’s Fund, Nairobi P.O. Box 44145-00100, Kenya; georgeku@unops.org (G.G.); fwrobert@unicef.org (F.W.); 8 Global Health Support Initiative III, Social Solutions International, Washington, DC 20523, USA; 9 Emergency and Resilience Division, Food and Agriculture Organization of the United Nations, 00153 Rome, Italy; abdalmonium.osman@fao.org (A.M.O.); darana.souza@fao.org (D.S.); 10 School of Economic Sciences, Washington State University, Pullman, WA 99164, USA; yoder@wsu.edu

**Keywords:** dietary diversity, arid and semi-arid lands, livestock for human health

## Abstract

Low dietary diversity is a key driver of undernutrition and remains a significant public health challenge in low- and middle-income countries. This study evaluated the effect of nutritional counselling and the provision of livestock feed, aimed at sustaining milk production during dry periods, on the dietary diversity of women and children in a pastoralist setting. **Methods:** A cluster randomized controlled trial was conducted among households in Laisamis subcounty, north-eastern Kenya, which were assigned to one of three arms: (1) an intervention arm providing livestock feed during critically dry periods, (2) an intervention arm providing livestock feed plus enhanced nutritional counselling (provided once a week, covering topics including hygiene and sanitation, breastfeeding, maternal nutrition, immunization and complementary feeding) or (3) a control arm. The dietary diversity of mothers and children was assessed every six weeks over two years. Panel difference-in-difference regression models were used to estimate intervention effects on dietary outcomes including child minimum dietary diversity (MDD), minimum acceptable diet (MAD), women’s dietary diversity (MDD-W) and food security. **Results:** A total of 1734 households participated (639 in arm 1, 585 in arm 2, and 510 in the control arm). The provision of livestock feed alone had significant gains in child MAD (OR 1.20; 95% CI 1.08–1.34), child MDD (OR 1.15; 1.11–1.20), and MDD-W (OR 1.10; 1.01–1.19) whereas combined livestock feed with counselling, reduced child food poverty (OR 0.89; 95% CI 0.81–0.99), increased child MAD (OR 1.39; 1.22–1.52), and improved MDD-W (OR 1.21; 1.16–1.28) relative to control. Neither intervention increased child minimum meal frequency relative to control. Purchasing livestock was associated with higher odds of meeting dietary-diversity indicators but a lower meal frequency (OR 0.80; 0.80–0.90); in contrast, cash-transfer receipt was linked to reduced odds of achieving child MDD (OR 0.90; 0.87–0.94), child MAD (OR 0.95; 0.85–0.97), and women’s MDD (OR 0.73; 0.54–0.89). **Conclusions:** Livestock feed provision sustains milk consumption and improves dietary diversity in pastoralist populations. When combined with nutritional counselling, these interventions strengthen the link between animal and human health, with important implications for food security.

## 1. Introduction

Undernutrition remains a significant public health challenge in sub-Saharan Africa, with women of reproductive age and children <5 years of age particularly affected [1]. Household sanitation, income, feeding, and caring practices have been identified as important determinants of undernutrition in these groups [2,3]. Globally, an estimated 820 million people were undernourished in 2020, and nearly 45% of deaths among children <5 years of age were linked to undernutrition [4].

Consumption of a diverse diet has been associated with a reduction in undernutrition (underweight, wasting, and stunting) among children <5 years old and women of reproductive age [5,6,7]. Dietary diversity is assessed by either evaluating the number of food groups consumed or quantifying the intake of micronutrients within each food group over a 24 h period. Guidelines have been established to classify the recommended food groups for children <5 years old and women of reproductive age, and both groups need at least five food groups to ensure adequate uptake of micronutrients [8,9].

In Kenya, the 2022 Demographic and Health Survey reported stunting, underweight, and wasting in 18%, 10%, and 5% of children <5 years, respectively [10]. A comparison with the 2014 survey results indicated an 8% reduction in stunting, a 1% decrease in underweight, and a 1% increase in wasting [11]. In both surveys, regions predominantly inhabited by pastoralist communities consistently recorded the lowest proportions of children meeting the recommended dietary diversity scores [10,11].

Poor dietary diversity scores and high undernutrition levels have been reported in pastoralist communities in sub-Saharan Africa [12,13,14,15,16]. These communities predominantly rely on animal-source foods for their nutritional needs, with milk consumption contributing up to two-thirds of the mean caloric daily intake among children [17]. However, this reliance is heavily contingent upon the availability of forage [17,18]. A significant increase in undernutrition is observed during the dry season, correlating with decreased pasture availability and reduced animal milk production [19,20]. The increased cost of alternative food sources such as cereals during this period also leads to a decline in energy intake [19].

Improving dietary diversity in pastoralist areas has been challenging due to factors such as climate variation, entrenched cultural practices, and limited access to diverse foods [21]. Diets comprise dairy products and maize, with little consumption of vegetables, fish, fruits, and eggs, which are culturally viewed as low-value food sources [22]. Promotion of contextualized feeding practices through nutritional counselling among vulnerable groups is recommended to improve dietary diversity in Kenya [23,24,25]. This strategy is also recommended in the Kenya national Maternal, Infant, and Young Child Nutrition (MIYCN) policy guidelines [26].

Despite extensive observational work linking dietary diversity to child and maternal nutrition in pastoralist settings, three gaps persist: First, most studies are descriptive or cross-sectional, limiting causal inference on which factors improve dietary diversity during climate-sensitive dry periods [12,13,14,15,16]. Second, interventions typically target behavior alone (nutritional counselling) or supply alone (food supplementation), with few evaluating integrated, context-specific packages that address the binding constraint in pastoralist households—seasonal milk scarcity driven by forage failure [17,18,19,23,24,25]. Third, there is scant evidence from the arid and semi-arid lands (ASALs) of Kenya that disaggregates impacts for women of reproductive age versus children under five or that tests seasonally timed delivery aligned with peak nutritional risk [27].

This study aimed to evaluate the influence of nutritional counseling and the provision of livestock feed to milking animals during critical dry periods on the dietary diversity of children and women of reproductive age. Through this investigation, we sought to generate local evidence about the importance of feeding practices among vulnerable groups reliant on animal-source food availability, which hinges on forage conditions.

## 2. Materials and Methods

### 2.1. Study Area

**Figure 1:**
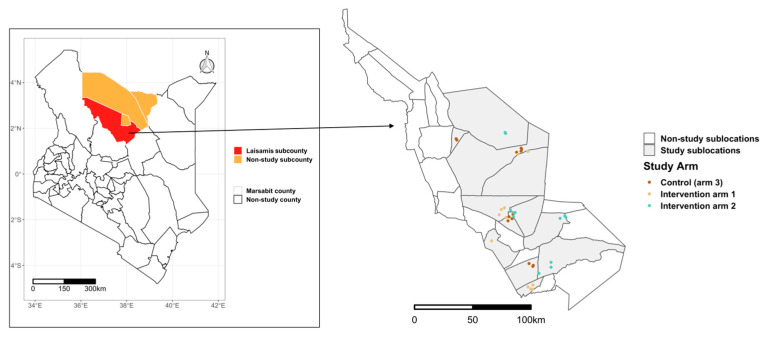
Map of Laisamis subcounty showing the selected sublocations and the villages in the different study arms. The inset map on the left shows the location of Marsabit County.

This study was conducted in Laisamis subcounty, Marsabit County, which is in the northern part of Kenya (Figure 1). The subcounty has reported the lowest measures of dietary diversity in children and the highest proportion of malnourished women compared to the rest of the subcounties in Marsabit [28].

The study utilized data from the Livestock for Health (L4H) Project, a cluster randomized control trial that aimed to investigate the effect of livestock supplementary feeding interventions during critical dry periods and enhanced nutrition counseling on maternal and child nutrition [29].

### 2.2. Data Sources

The study enrolled households in two intervention arms and one control arm, with approximately 600 households allocated to each arm. These study arms were spread across Laisamis subcounty, ensuring a minimum of five kilometers between villages in different arms to minimize the risk of contamination (Figure 1). In intervention arm 1, households received 0.35 tons of supplementary livestock feed adequate for two tropical livestock units (2 cows, 2 camels, 20 goats, or 20 sheep) for a duration of approximately 90 days in a dry season. Households in intervention arm 2 received supplementary livestock feed similar to arm 1 during the dry period, along with enhanced nutrition counseling sessions held once a week throughout the 2-year study period. These counseling sessions were informed by the Kenya Maternal, Infant, and Young Child Nutrition (MIYCN) policy guidelines [26]. Counselling was delivered face to face for a minimum of 30 min and covered key topics, including hygiene, breastfeeding, maternal nutrition, immunization, complementary feeding, growth monitoring, antenatal care, and milk handling and sanitation. The control arm did not receive any intervention during the study period, though animal feed for one dry season was provided at the end of the study.

In all three study arms, households with at least one child <3 years and a mother of reproductive age were recruited. Data were collected between September 2019 and December 2021, with children born during this period also enrolled in the study. The study design, including details on household selection and baseline results, have been published elsewhere [29].

### 2.3. Data Collection and Categorization

**Figure 2:**
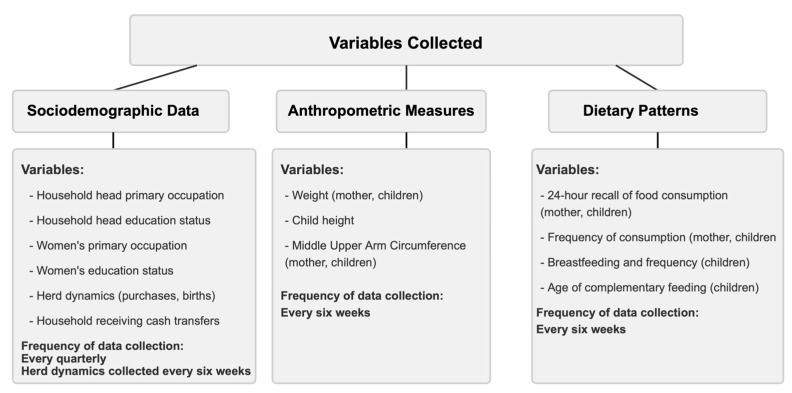
Overview of variables collected during each collection period.

Household socio-demographic data, individual-level demographic and anthropometric data, and 24 h recall data of dietary intake and frequency of consumption for both women and children were collected using a questionnaire administered to the caregiver. Figure 2 summarizes the variables collected and their frequency.

Infants <6 months: An analysis was conducted on the proportion of children exclusively breastfeeding and those who were breastfeeding with complementary feeding.

Children 6–23 months: The diet data were categorized into eight groups (Table 1) as per the WHO guidelines [9]. We computed the proportion of children who were breastfeeding, the reported age (in months) that complementary feeding commenced, dietary diversity score, meal frequency score, acceptable diet score, child poverty score, egg/flesh food consumption, and vegetable/fruit consumption. Children who consumed fewer than three food groups were categorized as experiencing severe child food poverty [30]. In addition, children with a dietary diversity score of ≥5 were categorized as having attained the minimum dietary diversity (MDD). Infants aged 6–8 months who had a meal frequency of at least twice a day, breastfeeding children aged between 9 and 23 months who had a meal frequency of at least thrice a day, and non-breastfed children who had a meal frequency of at least four times a day were categorized as having the minimum meal frequency (MMF). Children who had obtained both the minimum dietary diversity and minimum meal frequency for their age were categorized as having a minimum acceptable diet (MAD) (Table 1).

Children 24–59 months: The diet data were categorized into seven groups (Table 1). Similar to children aged 6–23 months, the dietary diversity score, meal frequency score, acceptable diet score, child food poverty score, egg/flesh food consumption, and zero vegetable/fruit consumption were calculated. Children were categorized as having the MDD and MMF if they consumed at least four food groups and had a meal frequency of at least four times a day, respectively (Table 1). Children who had obtained both the MMD and MMF for their age satisfied the MAD standard. Children who had consumed <3 food groups were categorized as experiencing severe child food poverty.

Women of reproductive age: The diet data were categorized into ten food groups (Table 1) based on the guidelines by the FAO and USAID’s Food and Nutrition Technical Assistance III project [8]. The MDD for women of reproductive age (MDD-W) was achieved if at least five of the ten food groups had been consumed.

### 2.4. Analytical Methods

For all study participants, panel estimators for generalized linear difference-in-difference regression models were used to estimate the effect of the interventions on the likelihood of a seasonal change in dietary diversity scores. We also investigated the association between indicators of feeding practices and the interventions provided in arms 1 and 2. Regressors in these models included gender (for children), the literacy status of women and household heads, the primary occupation of women and household heads, household participation in any social safety net program, herd dynamics (births and purchases), and herd health. The analysis was conducted using R version 4.4.2.

## 3. Results

**Figure 3:**
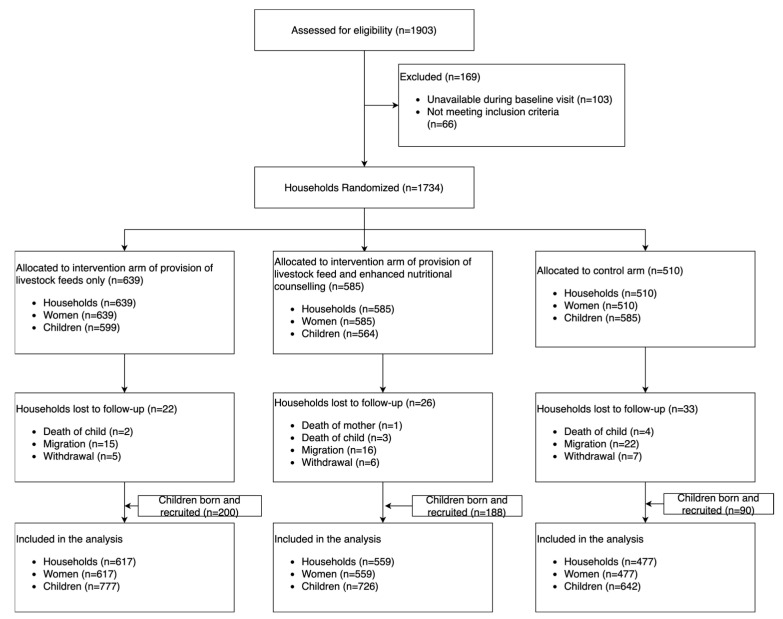
Participant flow diagram for the cluster randomized controlled trial.

A total of 1734 households with 1734 women and 1748 children were enrolled in the study and followed up every six weeks for a two-year period. During the baseline period, 1748 children were part of the study, categorized as either infants aged < 6 months (*n* = 269, 16%), children aged 6–23 months (*n* = 1009, 58%), or children aged 24–59 months (*n* = 367, 21%). Over the course of the study period, 81 households were lost to follow-up either because of migration (63%), withdrawal (24%), a child’s death (12%), or the mother’s death (1%) (Figure 3). A total of 478 children were born and recruited into the study during the follow-up visits.

### 3.1. Dietary Patterns

#### 3.1.1. Children

**Figure 4:**
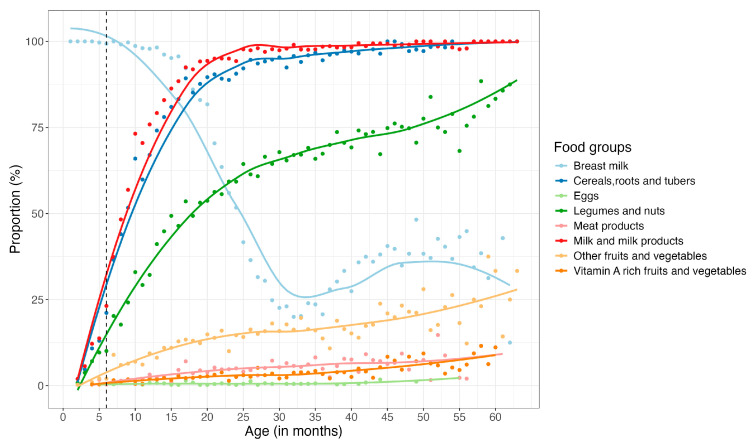
Food groups consumed by children <5 years over the study period. The dots represent the actual monthly proportion for each food group, whereas the line represents the trend line.

Within the first 6 months, nearly all children were breastfed, with this trend persisting until about the 18th month, at which point a sharp decline in breastfeeding was observed up until the 32nd month (3rd year), when less than 25% of the children were still consuming breast milk. Interestingly, an increase in breastfeeding was observed between the third and fourth years, followed by another decline (Figure 4). On average, mothers reported that complementary feeding began at six months of age. However, 35% of children had already started complementary feeding before this age. By the end of the first year, over 75% of children were consuming animal milk and milk products, cereals, roots, and tubers. Between 6 and 60 months, these foods groups remained predominant in the children’s diet, with an increasing trend observed in the consumption of legumes and nuts and fruits and vegetables (not rich in vitamin A) (Figure 4). Notably, throughout the study period, eggs, meat, and vitamin A-rich fruits and vegetables were the least consumed food groups by children <5 years (Figure 4). For a detailed breakdown of the proportions of the various food groups consumed during the study period, please refer to Appendix A Table A1 and Table A2.

#### 3.1.2. Women of Reproductive Age

Over the study period, women’s diets predominantly comprised only three of the ten recommended food groups: dairy products (92%); grains, roots, and tubers (90%); and pulses (68%). Less than one-fifth of the women consumed fruits/vegetables (8%), eggs (3%), and flesh foods (8%). No participant consumed nuts and seeds during the study period. **Appendix A** Table A3 provides the detailed consumption patterns among women.

**Figure 5:**
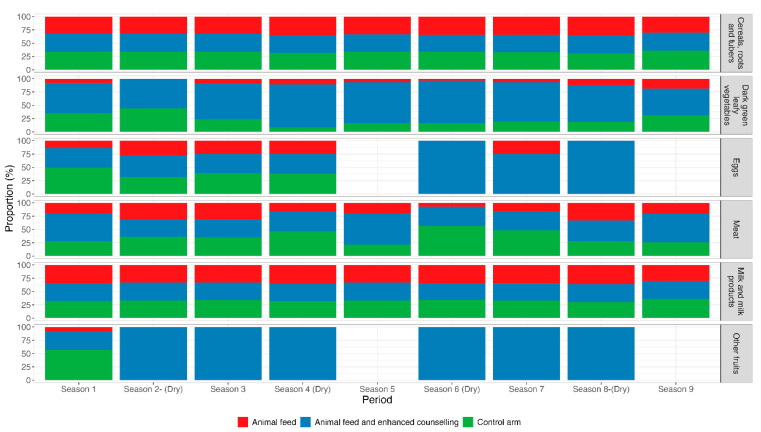
Proportion of women in the different intervention arms consuming each of the different food groups over the study period. The dry seasons occurred from January to March and July to October. Season 1 corresponds to September to December 2019, and Season 9 corresponds to November to December 2021.

Across the three intervention arms, the intake of cereals, roots, and tubers and dairy products remained consistent among women during both the dry and non-dry seasons. However, women in the intervention arm receiving animal feed combined with enhanced counselling reported a higher consumption of dark green leafy vegetables, fruits, and eggs (Figure 5). This group also reported a marked increase in egg consumption during the dry seasons.

### 3.2. Dietary Intake Indicators

**Figure 6:**
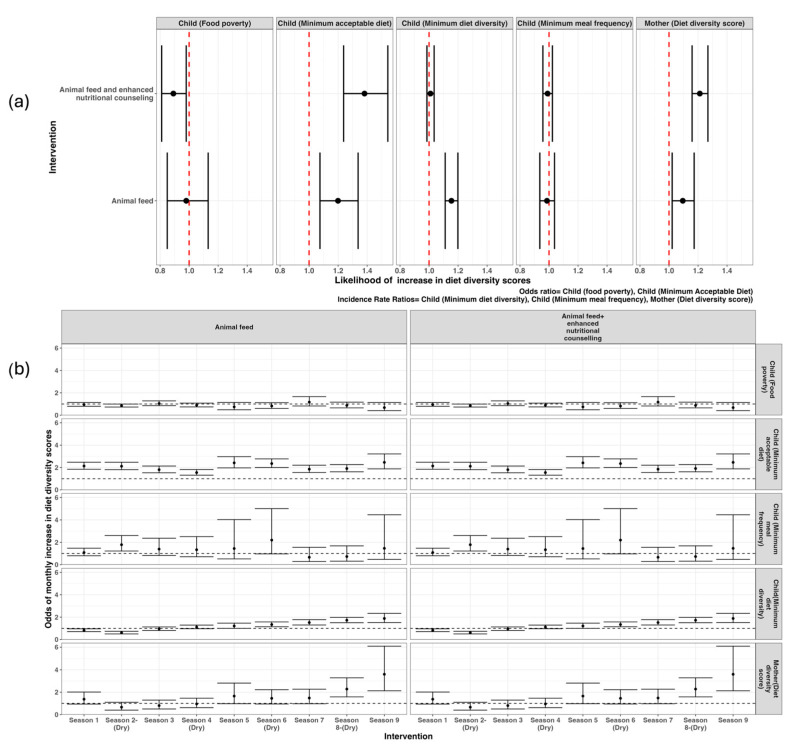
Likelihood of overall change in the indicators of dietary diversity score (**a**) and seasonal change over time (**b**). The dry seasons occurred from January to March and July to October. Season 1 corresponds to September to December 2019, and Season 9 corresponds to November to December 2021.

The difference-in-difference model results showed that, compared to the control group, households in arm 1 (animal feed only) were significantly associated with improvements in several child-specific indicators, namely, the minimum acceptable diet (OR 1.2; 95% CI 1.08–1.34) and minimum dietary diversity (1.15; 1.11–1.20), and in the mother’s dietary diversity score (1.10 [1.01–1.19]). Furthermore, the combined intervention of animal feed and enhanced nutritional counselling (arm 2) was significantly associated with a decrease in child food poverty (0.89; 0.81–0.99), an increase in child minimum acceptable diet (1.39; 1.22–1.52), and an improvement in the mother’s dietary diversity score (1.21; 1.16–1.28) (Figure 6a). Neither intervention had a significant effect on increasing the child’s minimum meal frequency compared to the control group (Figure 6a).

An analysis of dietary indicators over the study period showed no significant differences between the control group and the households receiving the different interventions in the baseline period. However, from the first month of the nutritional counselling intervention, there was a significant increase in the child dietary diversity score and minimum acceptable diet and a decrease in food poverty. Despite some fluctuations, the effect of the animal feed combined with enhanced nutritional counselling on the minimum acceptable diet was maintained throughout the study period (Figure 6b). Similar effects were observed in the mother’s dietary diversity score, which remained significantly higher from the first dry season (shaded in the gray areas in Figure 6b). The animal feed intervention significantly decreased child food poverty, increased the likelihood of children attaining the minimum acceptable diet, and improved the dietary diversity of both children and mothers during the dry periods (Figure 6b).

**Figure A1:**
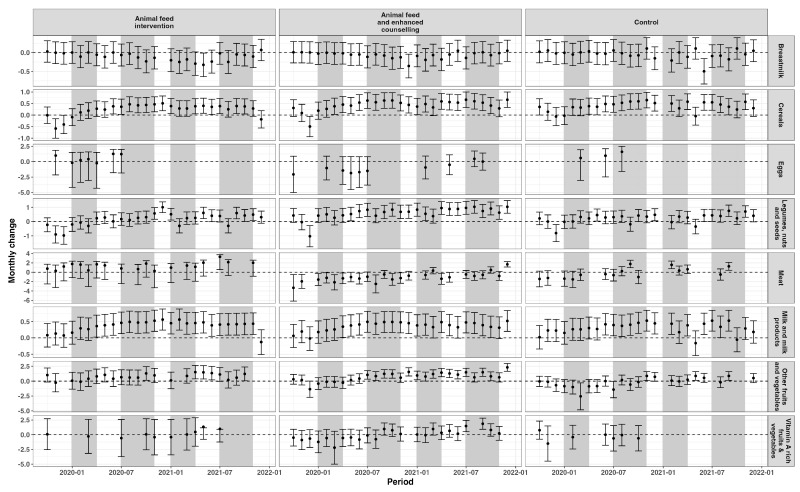
Likelihood of monthly change in the indicators of food groups over time for children aged 6–23 months in the three study arms (animal feed intervention, animal feed and enhanced counselling, and control arm). The grey shaded areas represent dry seasons.

**Figure A2:**
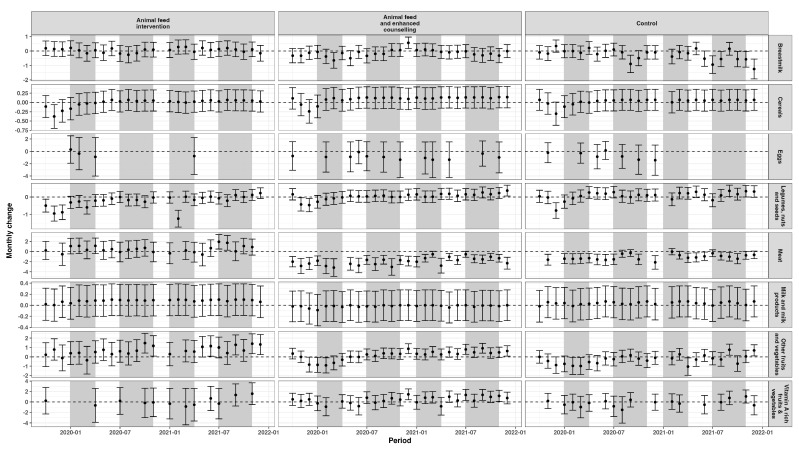
Likelihood of monthly change in the indicators of food groups over time for children aged 24–59 months in the three study arms (animal feed intervention, animal feed and enhanced counselling, and control arm). The grey shaded areas represent dry seasons.

**Figure A3:**
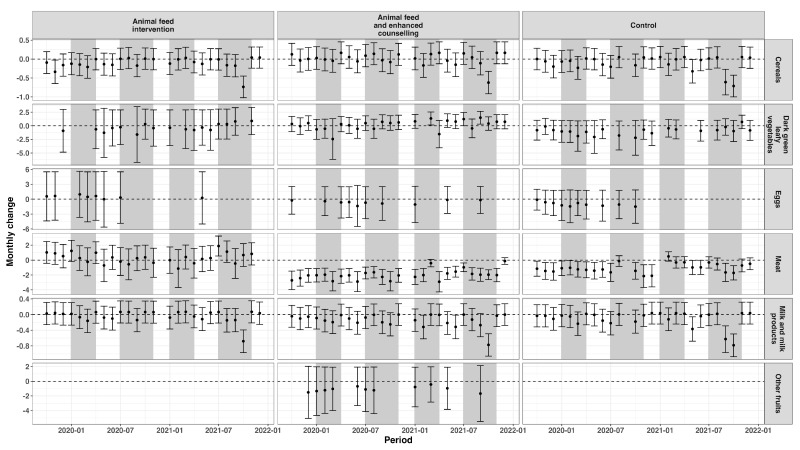
Likelihood of monthly change in the indicators of food groups over time for mothers of reproductive age in the three study arms (animal feed intervention, animal feed and enhanced counselling, and control arm). The grey shaded areas represent dry seasons.

**Figure A4:**
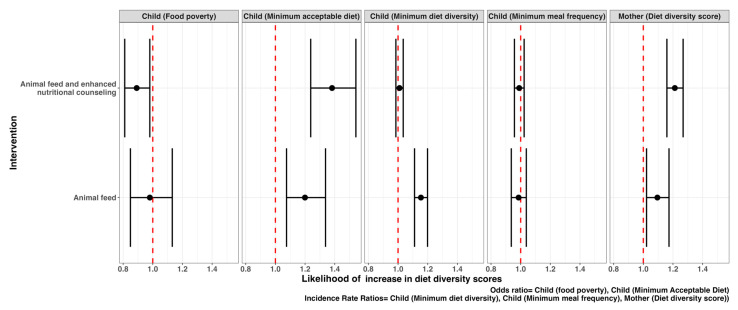
Likelihood of overall change in the indicators of dietary diversity scores. The vertical red dashed line marks the null value (OR = 1); estimates whose 95% CIs do not cross this line are statistically significant.

An analysis of the specific food groups showed a significant monthly increase in the consumption of cereals, legumes, nuts and seeds, and fruits and vegetables during the dry season among children aged 6–23 months within the households receiving animal feed with enhanced nutritional counselling in comparison to the baseline period (Appendix A Figure A1). However, milk consumption for children aged 6–23 months remained significantly high during the dry season for households receiving interventions, with the effect maintained throughout the study period. For the control group, milk consumption did not improve during the dry season in comparison to the baseline (Appendix A Figure A1, Figure A2, Figure A3 and Figure A4). Detailed results of the other food groups can be found in the Appendix A (Figure A1, Figure A2, Figure A3 and Figure A4).

### 3.3. Factors Associated with Feeding Patterns Among Households, Infants, Young Children, and Women of Reproductive Age

When controlling for other factors, the difference-in-difference regression results showed that both interventions were significantly associated with a higher likelihood of attaining the minimum dietary diversity scores (Table 2). The effect was particularly higher among households receiving animal feed combined with enhanced nutritional counseling, showing the following results: child MDD (OR 2.54; 95% CI 2.30–2.79), child MAD (3.10; 2.66–3.62), and women’s MDD (4.22; 3.29–5.42). Additionally, households with herd dynamics that included purchasing animals showed an increased likelihood of attaining the minimum dietary diversity scores but not the minimum meal frequency, which was significantly reduced by livestock purchases (OR 0.8; 95% CI 0.8–0.9) (Table 2). However, households benefitting from cash transfer programs had a significantly decreased likelihood of attaining the minimum scores for child MDD (0.90; 0.87–0.94), child MAD (0.95; 0.85–0.97), and women’s MDD (0.73; 0.54–0.89).

## 4. Discussion

This study assessed the effect of livestock feeding and nutritional counselling on dietary measures in infants, young children, and women of reproductive age in a pastoralist setting. The provision of animal feed either alone or in combination with nutritional counselling was associated with a significant increase in child minimum dietary diversity (MDD), minimum acceptable diet (MAD), and women’s minimum dietary diversity (W-MDD) and a decrease in child food poverty both overall and during the dry season. Enhanced nutritional counseling significantly increased the MDD and MAD and decreased child food poverty from the first month of intervention. The specific foods contributing to these improvements included cereals, legumes, nuts and seeds, milk and milk products, fruits, and vegetables. Milk consumption for children aged 6–23 months remained significantly higher in the intervention groups both during the dry season and throughout the study period. However, there was no significant increase in the consumption of meat, fish, and eggs in the intervention groups over the study period.

Sustaining milk consumption in human populations through the provision of animal feeds has also been documented in pastoralist settings in Ethiopia and Somalia [17]. The connection between animal health and human health is well established, with significant implications for food safety and security through the maintenance of healthy livestock [31,32]. Additionally, this link has economic and social impacts, influencing trade, income, and livelihoods in these regions, which have a low consumption of nutrient-dense foods [32,33].

High dietary diversity scores are positively associated with better nutritional status in both children and women [34,35]. However, these scores are particularly low in pastoralist settings due to the limited consumption of nutrient-dense foods, such as animal-source foods, fruits, and vegetables [14]. These communities primarily rely on milk, cereals, legumes, nuts, and seeds for their nutritional intake, a pattern observed in our study and corroborated by other studies in pastoralist areas [36]. The cultural context of pastoralist communities, where livestock is regarded as a sign of wealth, partly explains this dietary pattern [20].

Even with targeted nutritional counselling, the consumption of certain food groups, such as eggs and meat, remained consistently low. This reflects a combination of cultural, economic, and supply-side barriers. In many pastoralist settings, eggs and poultry are considered low-status foods, and cultural norms often discourage their consumption, particularly by women and young children [20]. Gender and age-based taboos, together with preferential allocation of certain animal source foods to men, further suppresses intake among women and children [37]. Economic constraints further limit household purchasing power during dry seasons, when the prices of cereals and alternative protein sources rise sharply, narrowing dietary choices [19]. Supply-side limitations are also critical: local production of small livestock and poultry is minimal, and households often prioritize livestock products, especially milk, for sale or herd sustenance rather than home consumption, with availability closely tied to forage conditions [17,18]. Taken together, these factors may explain why counselling alone may be insufficient to shift dietary practices, highlighting the need for integrated approaches that address cultural beliefs, household income, and local food availability [20,38].

Nutritional counseling has been observed to be an effective method for improving the intake of these foods, as evidenced both in our study and in other studies conducted in Malawi and Ethiopia [39,40,41]. This approach may be further enhanced by increased milk production; this contributes to income generation in the household, which may be used to purchase other food groups [42]. Women have been identified as a significant pathway to improving child nutrition outcomes, and their economic empowerment enhances the prioritization of food purchases for the household [43].

Interestingly, an increased proportion of children consuming breastmilk was observed among older children between three and four years of age. This trend may be explained by the practice of short birth intervals, which results in extended breastfeeding periods for older children [44]. However, despite the benefits of continued breastfeeding, the lack of dietary diversity in complementary foods can lead to nutrient deficiencies and increased vulnerability to malnutrition.

Cash transfer programs have been implemented in pastoralist communities to provide immediate financial relief, enabling households to purchase food and other essential items during critical dry periods [45]. Although this study found a negative association between cash transfers and high dietary diversity scores, the program has been shown to positively influence household nutrition, particularly benefiting women [46]. This negative association may be explained by the fact that social safety net programs already target the vulnerable populations that experience undernutrition, and longer-term investment is required to observe positive effects, or the cash transfers do not target women as the primary beneficiaries given their role in family feeding. To enhance effectiveness, cash transfers in ASAL settings could be adapted by aligning disbursements with dry seasons; linking transfers with nutrition counselling through community health units; or providing nutrition-sensitive vouchers for diverse, nutrient-rich foods where markets are functional.

Our recommendations align with Kenya’s Maternal, Infant, and Young Child Nutrition (MIYCN) strategy, which emphasizes dietary counselling, community health platforms, and cross-sector linkages to improve complementary feeding. Strengthening MIYCN in pastoralist ASALs could include seasonally timed nutrition counselling, delivered through community health units, alongside agriculture–nutrition interventions that stabilize access to animal-source foods during dry periods. Such integration would reinforce MIYCN’s focus on the minimum dietary diversity of women and children while addressing the unique supply constraints of pastoralist communities.

Limitations: We examined qualitative food consumption patterns in the preceding 24 h during the 2-year period but excluded quantitative food consumption data. Although these data are important in reflecting the dimensions of the dietary quality of micronutrient adequacy and reduce the chance of recall bias, qualitative data are usually used as a proxy for individual and household dietary diversity. Similarly, studies have reported a low probability of recall bias in 24 h dietary assessments using both quantitative and qualitative methods [47].

## 5. Conclusions

The provision of animal feed among pastoralist communities combined with enhanced nutritional counseling during critical dry periods is associated with a more than four times increase in the dietary diversity scores of women and a more than twofold increase in those of children <5 years. These findings highlight the value of seasonally responsive, integrated interventions that address both supply- and demand-side constraints to dietary diversity in arid and semi-arid lands. Nutritional counselling should not only reinforce recommended infant and young child feeding practices but also target shifts in community perceptions that limit the uptake of nutrient-dense foods, such as eggs, meat, fruits, and vegetables. Embedding these approaches within Kenya’s existing Maternal, Infant, and Young Child Nutrition (MIYCN) policy framework would provide a clear, actionable pathway for reducing malnutrition risk and strengthening resilience in pastoralist communities.

## Figures and Tables

**Table 1:** Food group categorization for the dietary diversity of the household, children, and women and the diet scores.

Food Group Number	Children (6–23 Months)	Children (24–59 Months)	Women
1	Cereals, roots, and tubers	Cereals, roots, and tubers	Cereals, roots, and tubers
2	Vitamin A-rich fruits and vegetables	Vitamin A-rich fruits and vegetables	Dark green leafy vegetables
3			Other vitamin A-rich fruits and vegetables
4	Other fruits and vegetables	Other fruits and vegetables	Other fruits
5			Other vegetables
6	Flesh foods	Flesh foods	Flesh foods
7	Eggs	Eggs	Eggs
8	Legumes, nuts, and seeds	Legumes, nuts, and seeds	Legumes
9			Nuts and seeds
10	Dairy products	Dairy products	Dairy products
11	Breastmilk	-	-
Total Food Groups	8	7	10
Diet Scores			
Minimum Dietary Diversity Score (MDD)	Consumption of at least 5/8 food groups	Consumption of at least 4/7 food groups	Consumption of at least 5/10 food groups
Minimum Meal Frequency (MMF)	6–8 months: At least 2 meals a day 9–23 months: At least 3 meals a day Non-breastfed children: At least 4 meals a day	At least 4 meals a day	-
Minimum Acceptable Diet (MAD)	Attainment of both minimum dietary diversity and minimum meal frequency		-

**Table 2:** Difference-in-difference regression results of the effects of factors associated with minimum dietary diversity (MDD), minimum meal frequency, and minimum acceptable diet in children and women of reproductive age.

		Infants and Young Children			Women of Reproductive Age
	Dependent Variables →	Minimum Dietary Diversity (MDD) Odds Ratio (95% CI)	Minimum Meal Frequency (MMF) Odds Ratio (95% CI)	Minimum Acceptable Diet (MAD) Odds Ratio (95% CI)	Minimum Dietary Diversity-Women (MDD-W) Odds Ratio (95% CI)
Factors ↓					
**Study arm**					
Control arm		Reference	Reference	Reference	Reference
Animal feed only		**1.78 (1.60, 1.98)**	**2.19 (1.56, 3.07)**	**1.77 (1.53, 2.04)**	**1.55 (1.23, 1.89)**
Animal feed and enhanced nutritional counselling		**2.54 (2.30, 2.79)**	**1.61 (1.08, 2.39)**	**3.10 (2.66, 3.62)**	**4.22 (3.29, 5.42)**
**Gender**					
Female		Reference	Reference	Reference	
Male		0.83 (0.67, 1.02)	1.19 (0.94, 1.51)	1.07 (1.00, 1.15)	-
**Household head primary occupation**					
Livestock herding		Reference	Reference	Reference	Reference
Non-livestock herding		**0.47 (0.42, 0.52)**	1.18 (0.69, 2.03)	**2.29 (2.02, 2.60)**	**2.81 (2.24, 3.54)**
**Household head education status**					
Received formal education		**2.33 (2.10, 2.60)**	1.53 (0.74, 3.16)	**0.47 (0.40, 0.57)**	0.93 (0.70, 1.24)
No formal education		Reference	Reference	Reference	Reference
**Women’s primary occupation**					
Livestock herding		Reference	Reference	Reference	Reference
Non-livestock herding		**1.75 (1.62, 1.89)**	**1.43 (1.09, 1.88)**	**1.21 (1.13, 1.30)**	**2.41 (1.93, 3.01)**
**Women’s education status**					
Received formal education		**3.02 (2.69, 3.38)**	**2.53 (1.19, 5.38)**	0.93 (0.78, 1,11)	**5.29 (3.91, 7.18)**
No formal education		Reference	Reference	Reference	Reference
**Herd dynamics**					
Births (count)		**1.002 (1.001, 1.004)**	1.01 (0.99, 1.02)	0.98 (0.95, 1.12)	1.21 (0.85, 1.66)
Purchases (count)		**1.03 (1.01, 1.06)**	**0.84 (0.76, 0.93)**	**1.02 (1.01, 1.07)**	**4.73 (2.53, 8.85)**
Cash transfer		**0.90 (0.87, 0.94)**	1.08 (0.93, 1.26)	**0.95 (0.89, 0.97)**	**0.73 (0.54, 0.89)**

→ denotes dependent outcomes; ↓ denotes predictors (factors) tested for association with those outcomes; Bold values indicate statistical significance at *p* < 0·05.

## Data Availability

The data used to generate this manuscript may be found in the following repository: https://github.com/cema-uonbi/L4H_diet_diversity (accessed on 18 August 2025).

## Appendix A

**Table A1:** Recommended food groups consumed by infants and children <60 months during the baseline period.

Food Group	Infants < 6 Months (*n* = 269)	Children 6–23 Months (*n* = 1009)	Children 24–59 Months (*n* = 367)
Breast milk (exclusive)	244 (90%)	74 (7%)	-
Breast milk (non-exclusive)	28 (10%)	1025 (93%)	122 (32%)
Breastfed and non-milk liquids	15 (6%)	-	-
Breastfed and animal milk	10 (4%)	-	-
Tubers, roots, and cereals	3 (1%)	613 (58%)	348 (92%)
Pulses/legumes and nuts	2 (1%)	371 (34%)	254 (67%)
Dairy products	3 (1%)	898 (82%)	357 (95%)
Flesh foods	-	29 (3%)	23 (6%)
Eggs	-	7 (1%)	3 (1%)
Vitamin A-rich fruits and vegetables	-	19 (2%)	10 (3%)
Other fruits and vegetables	1 (<1%)	93 (8%)	44 (12%)

**Table A2:** Recommended food groups consumed by children <60 months during the study period.

	Children 6–23 Months					Children 24–59 Months				
Food Group	Overall	Intervention Arm 1	Intervention Arm 2	Control	*p* Value	Overall	Intervention Arm 1	Intervention Arm 2	Control	*p* Value
Breastmilk	87.0%	84.6%	89.8%	86.6%	0.093	31.6%	29.9%	34.1%	30.9%	0.131
Dairy products	82.4%	82.3%	84.9%	79.9%	0.356	97.6%	97.6%	98.4%	96.8%	0.06
Tubers, roots, and cereals	78.4%	77.8%	79.9%	77.4%	0.821	94.7%	93.3%	94.8%	96.0%	0.423
Pulses/legumes and nuts	47.7%	45.9%	50.9%	46.2%	0.424	66.4%	61.8%	70.6%	66.6%	0.072
Other fruits and vegetables	15.7%	8.5%	25.4%	11.2%	<0.001	16.9%	9.2%	26.5%	14.4%	<0.001
Flesh foods	7.4%	2.7%	9.1%	10.4%	0.092	6.4%	4.3%	6.8%	8.2%	0.057
Vitamin A-rich fruits and vegetables	4.8%	1.4%	7.1%	2.1%	0.009	5.1%	1.2%	7.9%	3.3%	<0.001
Eggs	1.6%	1.0%	2.1%	1.2%	0.214	1.4%	1.1%	1.1%	2.1%	0.015
Eggs/flesh foods	7.7%	2.9%	9.9%	10.5%	0.066	6.7%	4.4%	7.2%	8.7%	0.018
Fruits/vegetables	16.5%	8.6%	27.1%	11.6%	<0.001	17.6%	9.5%	27.8%	14.9%	<0.001

**Table A3:** Recommended food groups consumed by women of reproductive age during the study period.

	Baseline	Study Period			
Food Group	Women (*n* = 1734)	Intervention Arm 1	Intervention Arm 2	Control	*p* Value
Dairy products	92.7%	92.8%	91.4%	91.3%	0.573
Grains, roots, and tubers	92.2%	89.8%	90.0%	90.6%	0.892
Pulses	72.8%	66.5%	70.8%	67.3%	0.166
Other vegetables	15.1%	10.7%	27.2%	13.2%	<0.001
Flesh foods	8.2%	5.5%	9.0%	8.7%	0.058
Dark green leafy vegetables	1.7%	6.0%	8.8%	3.2%	0.037
Other vitamin A-rich fruits and vegetables	1.0%	1.4%	3.7%	2.0%	0.002
Eggs	0.6%	1.0%	2.0%	7.1%	0.035
Other fruits	0.3%	1.2%	1.2%	1.7%	0.060
Nuts and seeds	0%	0%	0%	0%	
